# Theoretical investigation on structural, functional and epitope of a 12 kDa excretory-secretory protein from *Toxoplasma gondii*

**DOI:** 10.1186/1472-6807-12-30

**Published:** 2012-11-27

**Authors:** Yap Boon Wooi Tommy, Theam Soon Lim, Rahmah Noordin, Geita Saadatnia, Yee Siew Choong

**Affiliations:** 1 Institute for Research in Molecular Medicine (INFORMM), Universiti Sains Malaysia, 11800 Minden, Penang, Malaysia

**Keywords:** *Toxoplasma gondii*, Excretory-secretory protein, Homology modeling, Epitope prediction, Molecular docking

## Abstract

**Background:**

*Toxoplasma gondii* is an intracellular coccidian parasite that causes toxoplasmosis. It was estimated that more than one third of the world population is infected by *T*. *gondii*, and the disease is critical in fetuses and immunosuppressed patients. Thus, early detection is crucial for disease diagnosis and therapy. However, the current available toxoplasmosis diagnostic tests vary in their accuracy and the better ones are costly.

**Results:**

An earlier published work discovered a highly antigenic 12 kDa excretory-secretory (ES) protein of *T*. *gondii* which may potentially be used for the development of an antigen detection test for toxoplasmosis. However, the three-dimensional structure of the protein is unknown. Since epitope identification is important prior to designing of a specific antibody for an antigen-detection based diagnostic test, the structural elucidation of this protein is essential. In this study, we constructed a three dimensional model of the 12 kDa ES protein. The built structure possesses a thioredoxin backbone which consists of four α-helices flanking five β-strands at the center. Three potential epitopes (6–8 residues) which can be combined into one “single” epitope have been identified from the built structure as the most potential antibody binding site.

**Conclusion:**

Together with specific antibody design, this work could contribute towards future development of an antigen detection test for toxoplasmosis.

## Background

Toxoplasmosis is a disease caused by the intracellular protozoan parasite, *Toxoplasma gondii*. The disease is estimated to infect more than one-third of the world population [1-5]. Although the infection may show mild symptoms or asymptomatic [6], it can be fatal in an immunocompromised patient or the fetus whose mother acquired primary infection during pregnancy [7]. The life cycle of *T*. *gondii* can be divided into two phases, sexual and asexual phase. The sexual phase o the life cycle of *T*. *gondii* occurs only in cats (felids; the primary host). The asexual phase occurs in other warm-blooded animals (including humans) where it transmits through food contaminated with the feces of infected cats [8].

Due to the high prevalence of toxoplasmosis, especially in third world countries, disease diagnosis and therapy are important. There are a number of diagnostic methods available which include IgM-ELISA, IgG-ELISA, IgG avidity test, Western blots and PCR using body fluids and tissues [9]. Some of these methods are time consuming, expensive and vary in their accuracy to diagnose acute infection.

An earlier published work has led to the discovery of a low molecular weight, highly antigenic 12 kDa excretory-secretory (ES) protein from *T*. *gondii* which is of potential to be used as a diagnostic marker to detect active infection. The antigenicity of the protein could lead to the development of an antigen or antibody detection test [4]. ES proteins are also known to be better antigens in diagnostic systems such as ELISA compared to crude antigens or somatic antigens as detection sensitivity are improved [10]. It was also found to be more effective in the diagnosis of swine trichinosis, toxocariasis, and ornithobilharziosis [11-13].

Development of an antigen detection test requires identification of B-cell epitope involved in antibody recognition. In order to identify the epitope(s), we modeled the three-dimensional structure of the 12 kDa ES protein of *T*. *gondii*. The results showed that the 12 kDa ES protein has a thioredoxin-like backbone and consist of three potential epitopes which function as a “single” antibody binding site.

The 12 kDa protein was also analyzed for its molecular docking capacity with apoptosis signal-regulating kinase 1 (ASK1) using ZDOCK, PatchDock and FireDock. Docking results showed that the predicted epitopes scored as the best ranked complex.

## Results

### Protein structure prediction

The 12 kDa ES protein consists of 106 amino acids. The result of BLAST against non-redundant (nr) protein database showed that the ES protein has a sequence identity of 46-79% with thioredoxin protein family of *Neospora caninum*, *Plasmodium* spp and *Saccharomyces cerevisiae* (Table 1). From the BLAST search against PDB, five best templates were selected. The selected templates had sequence identities of at least 37%. Templates selected were 3F3Q [14], 2I9H [15], 3F3R [14], 2FA4 [16] and 2HSY [17]. All the templates were obtained from *S*. *cerevisiae* (Table 2).

**Table 1:** **Results from non-redundant protein database BLAST search of the *T. gondii*.12 kDa ES protein**

**Accession No.**	**Sequence identity/similarity (%)**	**Organism of origin**	**Title**	**E-values**
CBZ49590.1	79/96	*Neospora caninum*	Thioredoxin	1e-47
XP_001348719.1	49/79	*Plasmodium falciparum*	Thioredoxin	6e-26
XP_002260299.1	47/78	*Plasmodium knowlesi*	Thioredoxin	8e-25
XP_001615815.1	47/79	*Plasmodium vivax*	Thioredoxin	3e-24
NP_013144.1	46/69	*Saccharomyces cerevisiae*	Thioredoxin1p	4e-22

**Table 2:** **Templates from BLAST search against Protein Data Bank (PDB) for the three-dimensional structure construction of *T. gondii *12 kDa ES protein**

**Template (PDB id)**	**Sequence identity/similarity (%)**	**Organism of origin**	**Title**	**E-values**	**Crystallographic resolution (Å)**
3F3Q^14^	46/69	*Saccharomyces cerevisiae*	Chain A, Crystal Structure Of The Oxidised Form Of Thioredoxin 1	9e-25	1.8
2I9H^15^	46/69	*Saccharomyces cerevisiae*	Chain A, Nmr Solution Structure Of Thioredoxin 1	9e-25	NA
3F3R^14^	46/69	*Saccharomyces cerevisiae*	Chain A, Crystal Structure Of Yeast Thioredoxin1-Glutathione Mixed Disulfide Complex	1e-23	1.8
2FA4^16^	37/69	*Saccharomyces cerevisiae*	Chain A, Crystal Structure Of Oxidized Form	2e-20	2.4
2HSY^17^	37/69	*Saccharomyces cerevisiae*	Chain A, Solution Structure Of Thioredoxin 2	2e-20	NZ

*NA*: Not applicable.

Sequence identity above 30% is sufficient to be used as templates for homology modeling technique [18-20]. The selected templates mentioned above were appropriate, especially since the sequence length of the ES protein is only 106 residues. Results of secondary structure prediction from all prediction servers were similar in terms of the total number of β-sheets and α-helices for the ES protein. The locations of the β-sheets and α-helices predicted were also found to be similar. The only difference in results from the servers was by Prof Server where an α-helix was predicted to be a β-sheet instead (Table 3).

**Table 3:** **Secondary structure prediction from the sequence and secondary structure calculation from the built structure of the *T. gondii *12 kDa ES protein**

	1	6	11	16	21	26	31	36	41	46	51	56
**Sequence**	**MPVHH**	**VTTEA**	**QFKSL**	**IEENE**	**MVLVD**	**FYAVW**	**CGPCR**	**QVAPL**	**VEAMS**	**EKPEY**	**AKVKF**	**VKIDV**
Jpred	--BBB	B--AA	AAAAA	AAA--	-BBBB	BB---	---AA	AAAAA	AAAAA	AAA--	---BB	BBBB-
PORTER	---BB	B--AA	AAAAA	AA---	BBBBB	BB---	-AAAA	AAAAA	AAAAA	AA---	---BB	BBBB-
Prof	--BBB	BB---	AAAAA	AA---	BBBBB	BB---	---AA	AAAAA	AAAAA	AA---	--BBB	BBBBB
APSSP2	--BB-	-AAAA	AAAAA	A--BB	BBBBB	-----	-AAAA	AAAAA	AAAAA	A----	BBBBB	BB---
STRIDE	---BB	---AA	AAAAA	AA---	BBBBB	BB---	-AAAA	AAAAA	AAAAA	A----	--BBB	BBBB-
Built Model	---BB	---AA	AAAAA	AA---	BBBBB	BB---	-AAAA	AAAAA	AAAAA	A----	--BBB	BBBB-
	61	66	71	76	81	86	91	96	101	106		
**Sequence**	**DELAD**	**VAERE**	**EINAM**	**PTFKL**	**FKQGK**	**AVDTV**	**VGANA**	**ERVEE**	**MVKKH**	**L**		
Jpred	----A	AAAAA	-----	-BBBB	BB---	BBBBB	----A	AAAAA	AAAA-	-		
PORTER	---AA	AAAA-	-----	-BBBB	BB--B	BBBBB	B---A	AAAAA	AAAA-	-		
Prof	BBBBB	BBBBB	-----	BBBBB	BB---	BBBBB	----A	AAAAA	AAAA-	-		
APSSP2	-AAAA	AAA--	BBBBB	BBBBB	-BBBB	BBB--	--AAA	AAAAA	AA---	-		
STRIDE	--AAA	AAAAA	-----	-BBBB	BB--B	BBBBB	B---A	AAAAA	AAAAA	-		
Built Model	--AAA	AAAAA	-----	-BBBB	BB--B	BBBBB	B---A	AAAAA	AAAAA	-		

*B *= Beta Sheets.

*A *= Alpha Helices.

**Figure 1:**
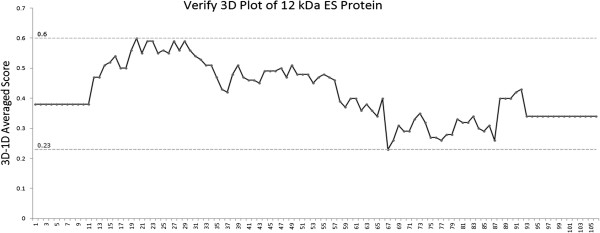
**Verify3D results for final built 12 kDa ES protein model. **All the residues 3D-1D score are higher than 0.2 with 0.23 as the lowest score of the residues.

Results from PROCHECK Ramachandran plot and verify3D showed that the best built model after energy minimization had 95.8% of the residues falling in the fully allowed region, 4.2% of the residues in the allowed region and 0% in generously allowed and disallowed regions. Results with > 90% of the residues in the fully allowed region and none falling on disallowed region indicates that the model is well built [21]. Verify3D test results showed that 100% of the residues had an average 3D-1D score higher than 0.2 which indicates a well built model since all the residues are valid in their folded conformation (Figure 1). PROCHECK evaluation showed −0.02 G-factor score for the 3D model of the ES protein. A model with an overall scores of G-factor higher that of −0.5 is accepted in general as an indication of a good quality model [21]. STRIDE result for secondary structure calculation also correlated well with the result of sequence based secondary structure prediction by the three servers mentioned above (Table 3).

**Figure 2:**
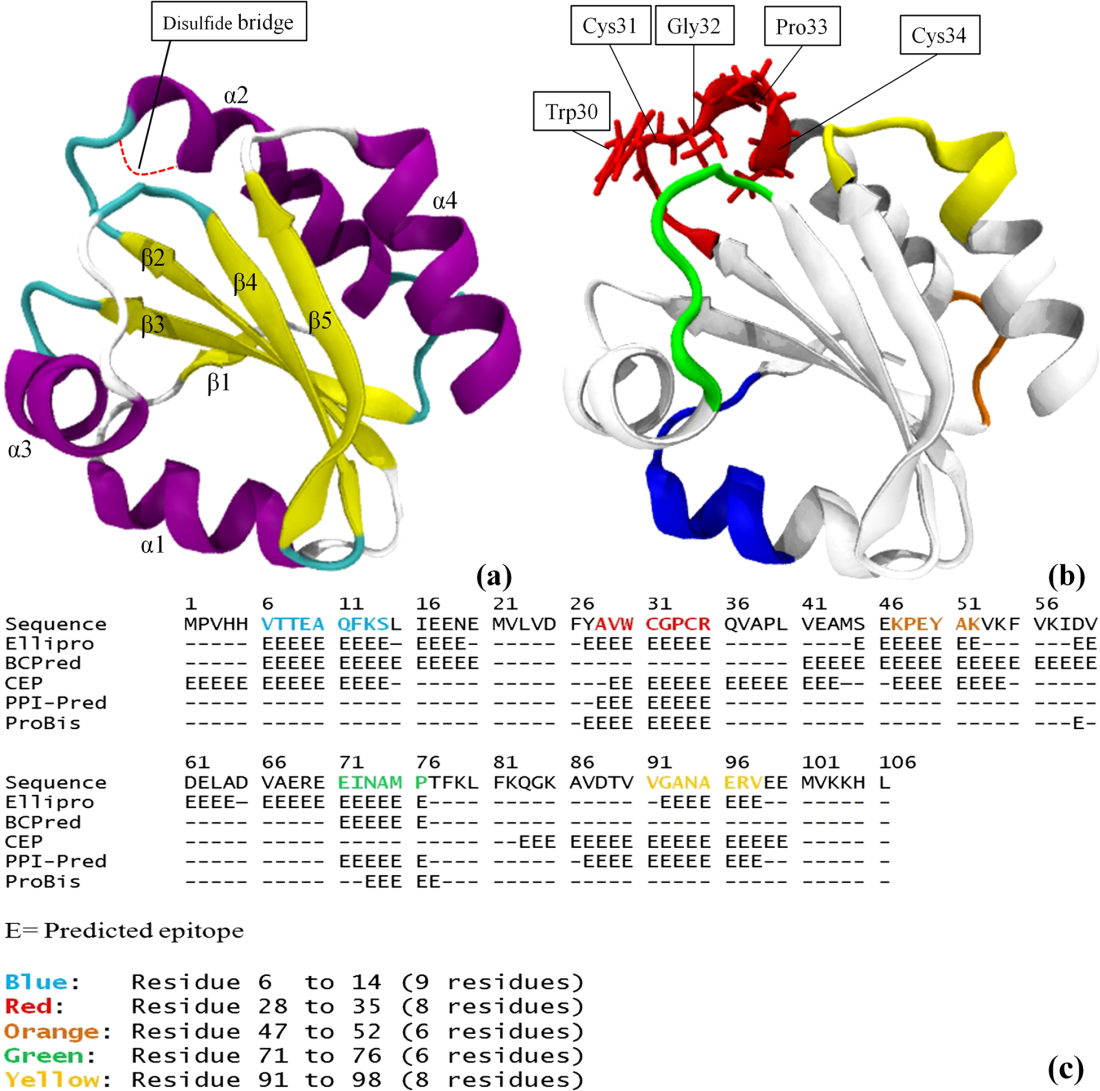
**Built structure of 12 kDa ES protein and it’s predicted epitopes. **(**a**) The 3-dimensional structure of the built 12 kDa ES protein model. Purple represents α-helices, yellow represents β-sheets, white represents coils and cyan represents turns. Disulfide bond between Cys31 and Cys34 is shown in red dotted line. (**b**) The predicted epitopes, represented in different colors as below (Figure 2c) of the *T. gondii *12 kDa ES protein. The conserved region Trp-Cys-Gly-Pro-Cys is in stick representation. (**c**) Summary of the epitope prediction results of the *T. gondii *12 kDa ES protein from four sequential and conformational epitope prediction servers and protein binding site prediction results from ProBis.

The 3-dimensional structure of the 12 kDa ES protein is an assembly of four α-helices (α1-α4) and five β-sheets (β1-β5) (Figure 2a). The β-sheets are flanked by the α-helices with all the loops and turns connecting the helices and sheets located on the surface of the structure. All the sheets are almost parallel to each other pairing with α2 and α4 while α1 and α3 are oriented perpendicularly.

### Epitope prediction

Epitopes of the ES protein were predicted using four different servers for both linear and conformational epitope predictions. Average accuracy of these prediction servers were reported to be 75% [22]. Epitope prediction servers showed comparable results. There were a few regions of the ES proteins that were predicted to be potential epitopes by at least three servers. Three out of the four servers predicting a region to be an epitope shows convincing result. From the result of the prediction, we can conclude that the five most possible epitopes are residue 6–14, 28–35, 47–52, 71–76 and 91–98 (Figure 2b &2c). Residue 91 was considered as one of the epitope residue even with only two epitope prediction servers predicting it to be an epitope because it is an important residue shown in previously published work [23]. Val91 is important because it might be involved in the binding of thioredoxin to other protein molecule due to its accessibility [23].

Protein binding site prediction server, ProBis, predicted that 2 of the above mentioned 5 epitopes to be the binding sites for ES protein. Results from ProBis showed that residue 27–35 and 73–77 are the protein binding sites.

### Molecular docking

**Figure 3:**
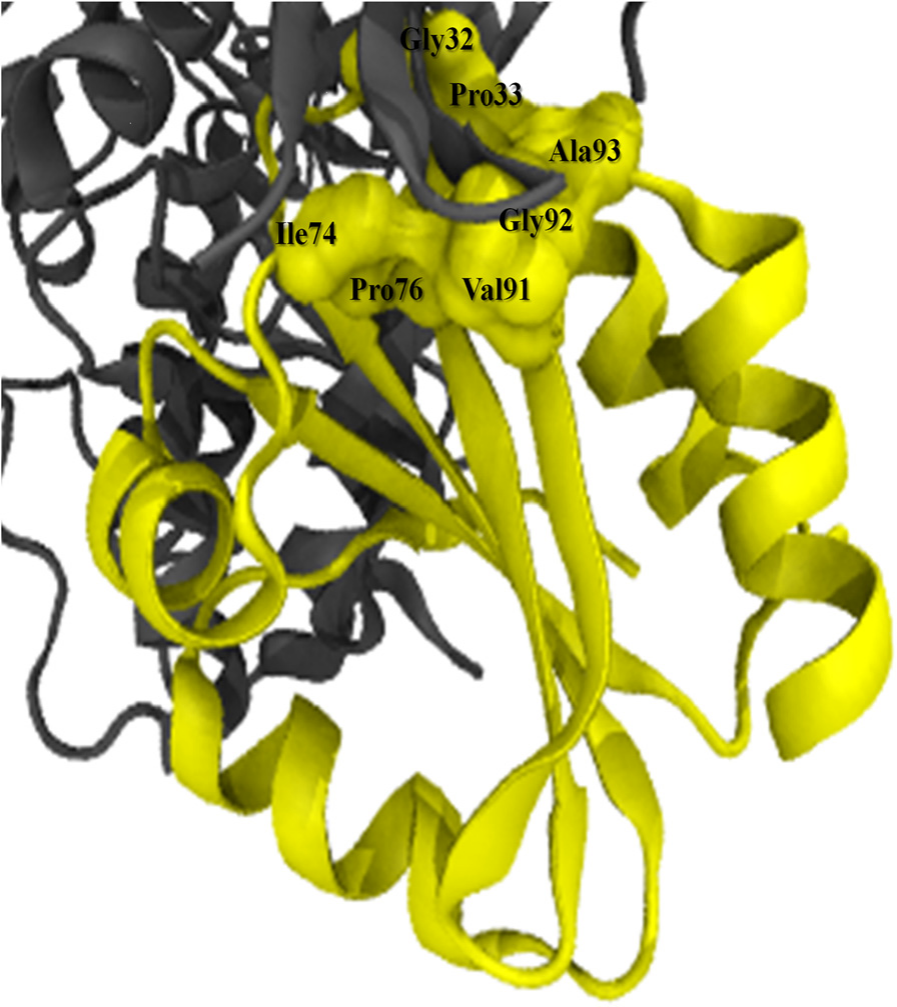
**The confirmation of the best ranked ASK1-ES protein complex from ZDOCK. **ASK1 and ES protein is in grey and yellow cartoon representation, respectively. The residues in ES protein which is important (Gly-32, Pro-33, Ile-74, Pro-76, Val-91, Gly-92, Ala-93) in the binding with ASK1 are in yellow surface representation.

ASK1 is a member of the mitogen-activated protein kinase kinase (MAPKKK) family. It is involved in the map kinase pathway where it activates certain kinase in response to stress [24]. A protein-protein docking simulation was performed by docking the modeled ES protein with ASK1 to further improve the confidence of the epitopes predicted from the ES protein. ASK1 was chosen as the docking partner of built ES protein as thioredoxin has been identified to be an ASK1-interacting molecule that plays an important role in oxidative stress-induced regulation of ASK1 [24]. Results from molecular docking of ES protein and ASK1 showed that our ES protein binds to ASK1. From ZDOCK, 2000 docking complexes were generated and were ranked from the best binding complex to the worst binding complex. The best ranked complex (Figure 3) showed that ES protein binds to the ASK1 on residue 28–35, 71–76 and 91–98. On the other hand, PatchDock provided results which were ranked according to a geometric shape complementarily score after molecular shape representation and surface patch matching [25]. These results were used for further refinement and re-scoring of 1000 top scoring complexes using FireDock [26,27]. The results from Firedock ranked the complexes according to the global energy score. The best ranked complex has a score of −25.62 which is generally good [26]. The complex showed that the ES protein was bound to ASK1 on the same residues as predicted by ZDOCK result (residue 28–35, 71–76 and 91–98). These epitopes were predicted by the epitope prediction servers as well as ProBis. Further analysis of the interaction between the ES protein and ASK1 shows spatial distances ranging between 4.3 Å and 8.4 Å from the important residues of ES protein (Gly-32, Pro-33, Ile-74, Pro-76, Val-91, Gly-92, Ala-93) with residues from ASK1.

## Discussion

Elucidating the structure of the 12 kDa ES protein from *T*. *gondii* is necessary to understand the functionality of the antigen. It is interesting to find that BLAST against nr protein database and PDB database, showed the 12 kDa ES protein has high sequence identity with thioredoxin proteins. It is the most abundant cellular-reducing dithiol catalyst which functions include redox regulation, protein folding, intracellular signaling and oxidative stress responses. The thioredoxin family is a large family of proteins consisting of domains that function biochemically by forming disulfide bonds with target molecules, resulting in conformational changes or rearrangement of disulfide bonds in the target. In cancer studies Trxs have been proposed as drug targets. Furthermore, components of the redox cycle have been considered targets in malaria parasites and trypanosomatids. The ES protein has also been identified to be mainly localized at the outer compartment of *T*.*gondii* apicoplast and it is discovered that an Apicoplast Thioredoxin-like protein 1 (ATrx1) was the first protein found to reside in apicoplast intermembrane spaces. Several enzymes found in the apicoplast that are potentially regulated by thioredoxin including 1-deoxy-d-xylulose 5-reductoisomerase, Clp protease and the protein translation factors EF-G and EF-Tu [4].

In secondary structure prediction study, all servers showed similar results except for Prof server. Prof server predicted a β-sheet (residue 63–70) instead of an α-helix, which contradicted with the other three servers. This contradiction could be mainly caused by the small size of the α-helix which consists of only 8 residues (residue 63–70). It was the shortest α-helix among all the α-helices in the built ES protein model. This secondary structure prediction can help in the verification for the tertiary structure of the built protein.

The built ES protein resembled thioredoxin proteins, consisting of four α-helices (α1- α4) flanking five mixed β-sheets (β1- β5) in the center of the protein (Figure 2a). Each α-helix and β-strand was connected through loops and turns. From the ES protein structure, all the loops and turns were located on the surface of the protein where they could be accessed easily and have a higher tendency to be the ES protein epitope. All four helices were also located on the surface flanking the β-sheets, causing the sheets to be buried in the center of the ES protein and thus less likely to be accessible. The β-sheets and α-helices can be subdivided into N- and C-terminal motifs which are connected by loops. The above mentioned orientation of the sheets, helices, loops and turns are similar to the thioredoxin protein family. Thus, this makes the 12 kDa ES protein a variant of thioredoxin [28].

Thioredoxin is a single-domain monomeric glutaredoxins-like protein with an extra β-sheet and α-helix at the N-terminal [28]. Thioredoxin exist either in a reduced form, with a dithiol, or in an oxidized form when its half-cystine residues form an intramolecular disulfide bridge [23]. The built model of the 12 kDa ES protein model is most likely in the oxidized form because of the presence of disulfide bridge linking two cystine residues (residue 31 and 34) (Figure 2a). Thioredoxin is also involved in reduction/oxidization (redox) reaction through the reversible oxidation of its active center dithiol to a disulfide and catalyzes dithiol-disulfide exchange reaction [23]. By having the backbone structure of a thioredoxin protein, the ES protein is expected to share similar functions with thioredoxins. The 12 kDa ES protein is made of 75% β-sheets and α-helices, which is similar in characteristic to thioredoxin protein, and making it exceptionally stable and highly resistant to heat [23]. Apart from that, the ES protein structure also has the Trp-Cys-Gly-Pro-Cys conserved region (Figure 2b). This region is possessed by thioredoxins and appears to be the location of the disulfide bridge linking the two cystine residues (residue 31 and 34) within the conserved region between the β2 and α2. This conserved region is found in residue 30 to 34 of the 12 kDa ES protein which is known to be the active site for redox activity [28].

Thioredoxin protein is ubiquitously expressed in all living cells, which has a variety of biological functions related to cell proliferation and apoptosis [29]. Thioredoxins are found to have anti-apoptotic effects and are identified as an interacting partner of ASK1. Study showed that residue Gly-32, Pro-33, Ile-74, Pro-76, Val-91, Gly-92 and Ala-93 of thioredoxin are involved in the binding of thioredoxin to other protein molecules [30]. From the results of the docking simulation performed between ES protein and ASK1, all of the above mentioned residues (ES protein has the similar residue number) are located in the binding site of ES protein and play a role in the binding and interaction of ES protein with ASK1. The expression of thioredoxin interacts with the N-terminal of ASK1 and inhibits ASK1 kinase activity and subsequent ASK1-dependent apoptosis. This interaction is highly dependent on the redox status of thioredoxin, indicating that the redox ability of thioredoxin is very important for apoptosis inhibition [31]. The molecular docking results also suggest that the ES protein is able to bind to ASK1, thus it can possibly inhibiting ASK1 kinase activity which could stop the mechanism of ASK1-dependent apoptosis.

Another study also suggested that mitochondrial thioredoxin is essential for cell viability and regulation of the mitochondrial apoptosis signaling pathway [29]. A novel thioredoxin reductase inhibitor study on human leukemia cell lines showed inhibition activity of cell growth and induction of apoptosis [31]. The inhibition of ES protein could also help in the inhibition of *T*. *gondii* proliferation which occurs in the tachyzoites stage. This is because cell proliferation of *T*. *gondii* and apoptosis occur in the host cells during *T*. *gondii* infection. Thus, inhibiting the ES protein could prevent cell apoptosis. This lead to the hypothesis that the ES protein may play an important role in the pathogenesis of *T*. *gondii* infection.

ES proteins are secreted by *T*. *gondii* to perform certain tasks such as invading its host cells or excreted as waste products into the blood circulation. Thus it could be useful for diagnostic purposes since it is expected to be present in blood of all actively infected patients [32]. In addition, previous studies showed that the diagnostic sensitivity and specificity of *T*. *gondii* ES antigens are higher than the crude parasite antigen by 5% and 6% respectively [10]. ES antigens are also known to improve the sensitivity of diagnostic tools compared to somatic antigens [11-13]. Thus, we conclude that ES antigens are potential biomarker candidates in the development of diagnostics for toxoplasmosis.

Epitope prediction is important to identify the binding site of an antigen which will interact with the antibody. Epitopes are usually located on the surface of an antigen to make it accessible to antibodies. In addition, epitopes also tend to be located on the loops and turns of an antigen [33]. Epitopes are predicted using the calculation of physiochemical properties of the residues and determining the residue’s antigenicity [34]. The results of epitope predictions in this study are consistent with the basic criteria of epitopes whereby most of the predicted epitopes are located in the loops and turns of the 12 kDa ES protein. The conserved region of thioredoxin seen in the ES protein, is one of the predicted epitopes. This region is actually conserved with Trp-Cys-X-X-Cys; where the X represents any amino acid. If the amino acids represented by the Xs vary in thioredoxins of different organism, the ES protein can be a very valuable biomarker as the conserved region will provide a very specific binding site for an antibody. Besides that, epitope can also be termed as “protein binding site”. Protein binding site of the ES antigen was predicted by ProBis [35-37], a protein binding site detection server which detected three of the predicted epitopes as the antigen’s binding sites, corroborating with the predicted epitopes.

An antibody does not necessarily binds to just one of the epitopes. It might bind to more than one epitope which are closely located in a folded protein but not in the linear sequence of an antigen. The epitopes of residue 27–35, 71–76 and 91–98 in ES protein may function as one “single” epitope to be bound with an antibody. Predicted epitopes can be linear and/or conformational depending on the binding mode with an antibody. All coloured region in Figure 2(a) are considered linear epitopes. However, if an antibody is bound to more than one coloured region, it can be considered as conformational epitopes. These predicted epitopes can thus be used in future design of a binder or an antibody (e.g. scFv) which is specific to the 12 kDa ES protein.

## Conclusions

A well-built 12 kDa excretory-secretory protein from *T*.*gondii* has been constructed via homology modelling approach. The 3-dimensional structure of the ES protein possesses the thioredoxin backbone and conserved region. The docking simulation also showed that this ES protein could have similar functions as thioredoxin protein. Epitopes and protein binding sites prediction from the built structure showed constructive location on where a specific binder could be designed to assist in the development of an antigen-detection test for toxoplasmosis.

## Methods

### Secondary structure prediction

The sequence of the 12 kDa ES protein from *T*. *gondii* was obtained from Genbank with the accession number of ADT65352. Secondary structure prediction was performed by four web-based programs namely Jpred [38], PORTER [39], Prof [40] and APSSP2 [41].

### Protein structure prediction

The sequence of the ES protein was used for Basic Local Alignment Search Tool (BLAST) [42] against non-redundant (nr) protein database to identify the protein class family. BLAST was also performed using PDB (protein data bank). The result from BLAST showed the availability of templates with > 30% sequence identity. Therefore, homology modeling technique was used to build the 3D structure of the 12 kDa ES protein. Homology modeling is a reliable method to generate a 3D model of a protein from its amino acid sequence. It requires at least one experimentally solved 3D structure called template that has a significant amino acid sequence similarity to the target sequence. Homology modeling and experimental structure elucidation complement each other [43]. A total of five templates were selected for homology modeling technique based on the sequence identities, sequence coverage, E-values and crystallographic resolution. Sequence alignment of ES protein and templates sequences was performed using MODELLER9v8 [43-46]. A total of 200 models were generated. Top 10 models from these 200 models were selected based on the best molpdf score and DOPE score. These selected 10 models were further energy minimized to release the steric collision of atoms. A total of 50 steps of steepest descent (SD) followed by 50 steps of conjugate gradient (CG) energy minimization were performed using Sander module from AMBER8 [47].

### Structure verification

All 10 minimized structures were evaluated for their backbone conformation and stereochemical properties by PROCHECK Ramachandran Plot [48]. Verify3D [49] was used to validate the protein in 3D form by scoring the possibility of each residue to exist in 3D form. Besides that, G-factor value was also taken into consideration in structure verification. The final model was selected based on the best result from both Ramachandran Plot and Verify3D. Secondary structure calculation on the final model was performed by STRIDE [50].

### Epitope prediction

Epitopes of the built 12 kDa ES protein were predicted using web-based sequential epitope prediction servers i.e. Ellipro [34] and BCPred [51,52]. Web-based conformational epitope prediction servers i.e. Ellipro [34], CEP [53] and PPI-Pred [54] were also used to predict the conformational epitopes from the built structure. ProBis [35-37], a protein binding site prediction swever which detects protein binding sites based on local structural alignment, was used to predict the protein binding sites of the ES protein for further confirmation of the epitopes.

### Molecular docking

The protein-protein docking between apoptosis signal-regulating kinase 1 (ASK1) (PDB ID: 2CLQ) [55] and ES protein was performed using web-based docking servers, ZDOCK [56], PatchDock [25,57] and FireDock [23,24]. All docking simulations were performed with the default parameters [25,26,56].

## Competing interests

The authors declare that they have no competing interests.

## Authors’ contributions

YBWT performed the work described in this paper and wrote the first draft of the manuscript. RN & GS provided the raw data and edited the manuscript. TSL participated in the experimental design and revised the manuscript. YSC designed the experiment, gave conceptual and technical advice, obtained the funding, coordinated the project and revised the manuscript. All authors read and approved the final manuscript.
